# Frequency of M287T/AS3MT Single Nucleotide Polymorphism in an Iranian Population

**Published:** 2017-01-01

**Authors:** Fatemeh Farhid, Fatemeh Nadali, Bahram Chahardouli, Saeed Mohammadi, Shahrbano Rostami, Kamran Alimoghaddam, Ardeshir Ghavamzadeh

**Affiliations:** 1 MSc, Department of Hematology, School of Allied Medical Sciences, Tehran University of Medical Sciences, Tehran, Iran; 2 Associate Professor, Department of Hematology, School of Allied Medical Sciences, Tehran University of Medical Sciences, Tehran, Iran; 3 Assistant Professor, Hematology-Oncology and Stem Cell Transplantation Research Center, Tehran University of Medical Sciences, Tehran, Iran; 4 PhD, Hematology-Oncology and Stem Cell Transplantation Research Center, Tehran University of Medical Sciences, Tehran, Iran; 5 Professor, Hematology-Oncology and Stem Cell Transplantation Research Center, Tehran University of Medical Sciences, Tehran, Iran

**Keywords:** AS3MT gene, Polymorphism, Arsenic

## Abstract

**Background** **:** To determine the frequency of the single nucleotide polymorphism M287T in exon 9 of the AS3MT gene in Iranian population and to assess the difference in allele frequencies with other ethnicities.

**Subjects and Methods** **:** Genotyping analysis was performed on 150 healthy subjects using the PCR-RFLP assay. We used chi-square analysis to check the deviation from Hardy–Weinberg equilibrium and compare of the observed genotype frequencies in various ethnic. The level of statistical significance was considered as p<0.05.

**Results** **:** The homozygous CC, homozygous TT and heterozygous CT genotypes were observed in 2%, 80% and 18% of participated individuals. The SNP rs11191439 passed the Hardy-Weinberg equilibrium chi-squared test with p-value>0.05 and had a minor allele frequency (MAF)>5%.

**Conclusion**: Iranians are genetically very similar to Caucasian and African individuals and they are considerably different from other East Asians including Koreans, Chinese and Japanese individuals. Due to genetic polymorphisms can contribute to the variability in AS3MT activity; they may contribute to interindividual as well as intra-ethnic differences in response to the detoxification of arsenic**.**

## Introduction

 Arsenic compounds are known to have variable adverse health effects including lung, bladder, kidney and skin cancer and other skin lesions.^        1 ^ Because most organic and inorganic arsenic compounds are without color, odor or taste; so, food, water or air contaminants by its composition are not easily identifiable. Hence exposure to Arsenic compounds continues in the normal population.^   2 ^ The concentration of Arsenic in food, air and soil varies widely. Also, its concentration is different in various regions of the world.^        3 ^

The general human population may be at risk of developing adverse health effects as the result of environmental contamination.^        1 ^ Arsenic concentration in the body is affected by various factors such as age, sex,^        4 ^ smoking,^   5 ^ diet.^   6 ^ Also genetic factors such as polymorphisms in genes related to arsenic metabolism play an important role in determining Arsenic concentrations in the body.^        7 ^ Previous studies reported that single nucleotide polymorphisms (SNPs) of the genes involved in Arsenic metabolism was associated with in vivo metabolite concentrations.^        8 ^ Therefore, genetic predisposition can play an important role in the body burden of Arsenic.

Arsenite methyltransferase )AS3MT( is one of the key enzymes involved in the transfer of a methyl group from S-adenosyl-L-methionine to trivalent arsenical and play a critical role in arsenic detoxification. As2O3 polymorphism at position 287 (Met287Thr) critically influences arsenic metabolism. The frequencies of Met287Thr, T>C (rs11191439) AS3MT gene have been studied in other ethnic groups of East Asia include: Chinese, Japanese, Korean,^        9 ^ Bangladesh^        8 ^, South Americans,^        10 ^ also Caucasian and African Americans but no data are available for Iranian population. The aim of this study was to determine the frequency of the single nucleotide polymorphism M287T (rs11191439) in exon 9 of the AS3MT in Iranians and to assess the difference in allele frequencies with other ethnicities.

## SUBJECTS AND METHODS

**DNA samples and genotyping of the rs11191439 SNP**

Informed consent was obtained from all participating individuals in this study. The genomic DNAs of 150 healthy subjects were extracted from peripheral blood, using standard salting out procedure. Polymerase chain reaction-restriction fragment length polymorphism (PCR-RFLP) method was used to genotype AS3MT, with the appropriate primer set (Table 1). Briefly, amplification was performed in a 25 µl reaction mixture using Taq DNA polymerase master mix RED (Ampliqon, Denmark), 20-40 ng of template DNA and 0.5 µM of each primer. The protocol of PCR reaction was consisted of initial denaturation 95 0C for 3 min followed by 30 cycles of denaturation at 94^o^C for 30s, annealing at 60^o^C for 30s and extension at 72oC for 30s followed by a final extension of 72 ^o^C for 5 min. After PCR reaction, the PCR product was affected by HpyCH4IV (restriction enzyme) and incubated at 37^0^C for 16 hours.

The digested products were then subjected to electrophoresis on a 2.5% Agarose gels.

**Table 1:** Primers for the region harboring the M287TSNP

**Primers**	**Sequence**
Forward Reverse	5` GTGCTGGAGATGAACCGTGAA-3` 5`-GCAAGGGCAAGAGCAGAAAGA-3`

**DNA sequencing analysis**

DNA sequence analysis was used to confirm the results of AS3MT genotyping by PCR-RFLP in 3 of each genotype. Briefly, cycle sequencing used the same primers as for PCR. The cycle sequencing reaction used the Big Dye Terminator v3.1 Cycle Sequencing kit (Applied Biosystems, Foster City, CA, USA) according to the manufacturer's instructions. Unincorporated ddNTP and residual contaminants were removed with the XTerminator kit (ABI) and samples were sequenced on the ABI 3130 genetic analyzer (Applied Biosystems).

**Statistical analyses**

The statistical analysis was performed using the Statistical Package for the Social Sciences (SPSS) version 16.0. The genotype and allele frequencies were determined by direct gene count method. Deviation from Hardy–Weinberg equilibrium and comparison of the observed genotype frequencies in various ethnic were tested using chi-square analysis. The level of statistical significance was considered as p<0.05.

## Results

 One hundred and fifty healthy individuals were enrolled in this study. Among the participants, the average age was 38 years and the age range was 15-65 years. Seventy-six (50.7%) were males and 74 were females (49.3%). Figure 1A shows the genotype results for AS3MT/M287T polymorphisms. The amplified fragment length was 232 bp. The PCR products were digested by restriction enzyme HpyCH4IVI and the AS3MT/Met287Thr, T>C (rs11191439) polymorphism was determined. The analyzed genotype information obtained was as follows: wild-type homozygous T/T (232 bp), heterozygous T/C (232 bp, 154 bp, 78 bp), homozygous mutant C/C (154 bp, 78 bp) (Figure 1A). Two samples representing each genotype were selected for direct sequencing. The results of PCR-RFLP analysis were consistent with those from sequence analysis (Figure 1B). Twenty-seven (18%) and 120 (80%) of individuals were heterozygous CT and homozygous TT, respectively (Table 2). We found 3 homozygous (2%) for the CC genotype in our sample population. The SNP rs11191439 passed the Hardy-Weinberg equilibrium chi-squared test with p-value>0.05 and had a minor allele frequency (MAF)>5%. Stratification of the population by gender revealed no significant differences in allele frequency between the genders (p=0.1).

The frequency distribution of AS3MT (rs11191439) different allele and genotypes in Iranian and other populations is shown in Table 3. Significantly different allele frequency was seen the SNP between Iranian and East Asians, including Koreans,^8^ Chinese^        9 ^ and Japanese^        11 ^ (p-value=0.00, Table 2).

**Figure 1A:**
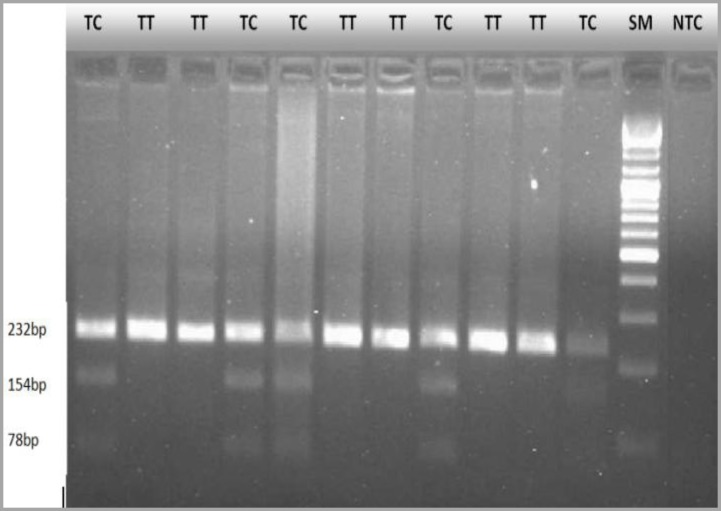
The genotype results for AS3MT/M287T polymorphisms

**Figure 1B:**
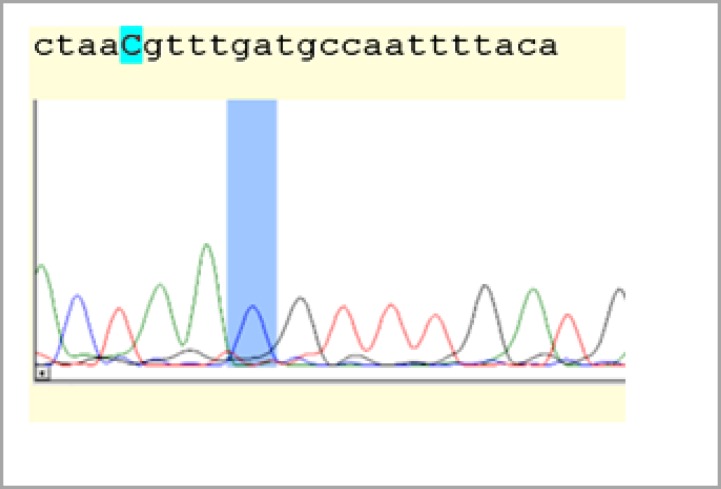
Sequence analysis of CC genotype

**Table 2:** Distribution of AS3MT/M287T genotypes among Iranian populations

**Characteristic**	**T; T (n=120)**	**C; T (n=27)**	**C; C (n=3)**
Age, median (Range)	38 (15-65)	37.9 (16-65)	22.3 (16-34)
Gender Male (n) Female (n)	57 64	18 8	1 2

The Iranian population also differed significantly from the other Asian population like Turk in the distribution of the M287T SNP.^        11 ^

There were no statistically significant differences observed in the distribution of T and C genotypes among the Iranian, Europeans, ^12^^,^^13^  African‐Americans and Caucasian Americans.^        14 ^

## Discussion

 Genetic polymorphisms are one of the important factors contributing to inter-individual variability in response to drugs. There are different intronic^        15 ^ and exonic^        12 ^ polymorphisms in AS3MT. From those variants, we selected M287T polymorphism because the effect of intronic polymorphisms on the AS3MT activity is still completely unknown and also previous studies have been shown to be associated with interindividual variation in the arsenic metabolism**.** It was first noted by Drobna et al. that genetic polymorphisms in AS3MT can influence DNA methylation in cultured primary human hepatocytes.^        16 ^ They showed that there was an association between the C allele in M287T and increased production of monomethyl arsenicals at medium.^        16 ^

To our knowledge, this is the first report evaluating the frequency of AS3MT/Met287Thr polymorphisms among Iranian individuals. We have defined ethnic differences in frequency of the Met287Thr SNP. We found that, the frequency distribution of Met287Thr was different from other reported populations in Asia.

Almost 10% of the European is heterozygous for the variant T allele compared with 1% of the East Asian populations.^        11 ^ A study in 2007 that examined a central European population, identified the M287T (T/C) polymorphism in the AS3MT gene is one of the major factors that influence arsenic metabolism,^        12 ^ a finding that has been confirmed in many populations including Bangladesh,^        8 ^ Chile^        17 ^ and Vietnam.^        15 ^

**Table 3:** Interethnic differences in the allele frequencies of AS3MT polymorphism by ethnic group

**Population**	**No of** **Individuals**	**Allele Frequencies**		**Ref**
		T	C	
**Japanese**	370	0.990	0.010	[11]
**Korean**	200	0.990	0.010	[11]
**Turk**	191	0.990	0.010	[11]
**Caucasian–** **American**	60	0.900	0.100	[14]
**African–** **American**	60	0.892	0.108	[14]
**Central ** **European**	411	0.891	0.109	[12]
**Polish**	201	0.875	0.125	[13]
**Present Study**	150	0.89	0.11	

One study of 17 populations showed substantial differences in 287T allele frequencies in Xhosas (0.233) compared with other population groups. In this study, similar frequency (above 0.100) of the 287T allele was reported in evaluating other African, Caucasian, African-American and Latin American populations.^        18 ^ The lower frequency of the 287T allele (ranging from 0.000 to 0.041) in Asian populations was reported in this study.^        18 ^ The lower frequency of AS3MT genotypes conferring higher enzyme activity may have a genetic advantage for individuals in more polluted areas.^        19 ^

Iranians are genetically very similar to Caucasian American, African American and central European population individuals and they are considerably different from other East Asians including Koreans, Chinese and Japanese individuals.^        12 ^ This result was consistent with the ethnic differences in previous studies on SNPs associated with drug metabolism,^        20 ^ phylogenetic.^   21 ^

Over the last decade, there has been a growing interest in the therapeutic potential of As2O3 as either single agent or in combination with ATRA for successful treatment of Acute Promyelocytic Leukemia (APL) patients.^22^^,^^23^ Awareness of such ethnic variations in the distribution of this polymorphism, characterization of AS3MT gene and the utilization of pharmacokinetic testing for the identification of different AS3MT alleles in patients may provide a useful tool for optimizing therapy and side-effects for the individual patient. Further studies are needed to assess the relationship between genetic variations and clinical outcomes in these patients.

## CONCLUSION

 In summary, we have demonstrated that Iranians have a significant increase in the frequency of the variant T allele when compared to other Asian ethnic groups that may underlie differential susceptibility to Arsenic toxicity. Over the last decade there has been a growing interest in the therapeutic potential of As2O3 as either single agent or in combination with ATRA for successful treatment of Acute Promyelocytic Leukemia (APL) patients.^22^^,^^23^ Awareness of such ethnic variations in the distribution of this polymorphism, characterization of AS3MT gene and the utilization of pharmacokinetic testing for the identification of different AS3MT alleles in patients may provide a useful tool for optimizing therapy and side-effects for the individual patient. Further studies are needed to assess the relationship between genetic variations and clinical outcomes in these patients.
